# The FaesMYB15-FaesELF3 module regulates flowering time, floral organ number, and floral color in red flower long-homostyle common buckwheat

**DOI:** 10.3389/fpls.2026.1944721

**Published:** 2026-10-28

**Authors:** Yalun Zheng, Haonan Liu, Yuzhen Liu, Zhixiong Liu, Zehao Hou

**Affiliations:** 1 College of Horticulture and Gardening, Yangtze University, Jingzhou, Hubei, China; 2 Hubei Key Laboratory of Spices &Horticultural Plant Germplasm Innovation & Utilization, Yangtze University, Jingzhou, Hubei, China; 3 Hubei Key Laboratory of Waterlogging Disaster and Agricultural Use of Wetland, Yangtze University, Jingzhou, Hubei, China

**Keywords:** FaesELF3, FaesMYB15, *Fagopyrum esculentum*, floral development, flowering time

## Abstract

EARLY FLOWERING 3 (ELF3) is a core member of the Evening Complex (EC) and plays a critical role in regulating flowering through the plant circadian clock. R2R3-MYB transcription factors (TFs) control the transcriptional regulation of anthocyanin structural genes involved in anthocyanin accumulation in various plants and also play important roles involved in floral development. However, how ELF3 homologs work together with R2R3-MYB TFs to regulate flowering, floral organ development, and flower color remains unclear. In this study, R2R3-MYB TF FaesMYB15 directly binds to the *FaesELF3* promoter and suppresses expression of *FaesELF3* in common buckwheat. In addition, overexpression of *FaesELF3* in red flower LH *Fagopyrum esculentum* leads to significantly earlier flowering and rapid plant growth, and results in flowers with increased tepal and stamen numbers and a lighter tepal color in transgenic plant. Moreover, total anthocyanin content in the inflorescence of the 35S::FaesELF3 line was significantly lower than that of the normal red flower LH common buckwheat. *FaesELF3* overexpression markedly downregulated the expressions of two core circadian clock genes, *FaesPRR5–1* and *FaesPRR5-2*, and two *AGAMOUS*-like MADS-box genes, *FaesAGL18* and *FaesAGL19*, involved in flowering regulation in transgenic plants. Our study introduces a new regulator module of FaesMYB15-FaesELF3 involved in regulating flowering time, floral color, and floral organ number in crops, providing insights for novel breeding strategies of flowering regulation and flower color modification in common buckwheat.

## 1 Introduction

The transcription factor (TF) EARLY FLOWERING 3 (ELF3) plays a critical role in the circadian rhythmicity machinery of plants (Zhu and Wang, 2024). ELF3 is a core member of the Evening Complex (EC), together with EARLY FLOWERING 4 (ELF4) and LUX ARRHYTHMO (LUX), which serves as a vital transcriptional repressor complex and constitutes a fundamental component of the plant circadian clock (Silva et al., 2020). In *Arabidopsis*, the transcriptional repressor AGAMOUS-LIKE 6 (AGL6) promoted floral transition by directly repressing *ELF3* and directly suppressing the expression of the floral integrator *FLOWERING LOCUS T* (*FT*), thereby regulating flowering through the FT-dependent pathway (Lee et al., 2024). *Arabidopsis* clock component *ELF3* not only serves as a light Zeitnehmer but also functions as a thermosensor participating in thermomorphogenesis (Zhu et al., 2022). Elevated salicylic acid (SA) levels exhibit significantly reduced thermoresponsive hypocotyl elongation and enhance *ELF3* binding to the *PHYTOCHROME-INTERACTING FACTOR 4* (*PIF4*) promoter at warm temperatures, and in turn decrease warmth-induced expression of *PIF4*, a central regulator of thermomorphogenesis, and of downstream auxin biosynthesis and signaling genes (Chen et al., 2025). *PSEUDO-RESPONSE REGULATOR 5* (*PRR5*), *TIMING OF CAB EXPRESSION 1* (*TOC1*), and the EC component ELF3 independently repress nighttime hypocotyl elongation via repressing *PIF4* expression in *Arabidopsis* (Won et al., 2024).

In rice (*Oryza sativa*), ELF3-like OsELF3 participates in the EC (OsEC) to regulate photoperiodic flowering and vegetative growth through interactions with OsELF4 and LUX (Cai et al., 2022). Moreover, OsELF3-1, OsELF4s, and OsLUX form an OsEC and then directly repress the expression of *Grain number*, *plant height*, *and heading date7* (*Ghd7*) (Sun et al., 2024). Ghd7 directly suppresses the expression of *ABC1 REPRESSOR1* (*ARE1*), a negative regulator of nitrogen use efficiency, thereby enhancing nitrogen uptake in rice (Sun et al., 2024). In wheat (*Triticum turgidum*), mutation of single *ELF3* homoeologue *TtELF3* resulted in obvious later heading than *Ttelf3*-null and significantly earlier heading than *TtELF3*-WT, which suggested that modified *TtELF3* function can affect heading date (flowering time) without disrupting circadian oscillations (Wittern et al., 2023). Wheat ELF3 operates downstream of *PHYTOCHROME B* (*PHYB*) as a direct transcriptional repressor of PHOTOPERIOD1 (PPD1), and that regulates expression of the florigen gene *FLOWERING LOCUS T1* (*FT1*) to regulate photoperiodic heading time in wheat (Alvarez et al., 2023). In addition, ELF3 homoeologue also plays an important role for the circadian clock in controlling the timing of senescence and grain nutrition in bread wheat (Buckley et al., 2025). Moreover, wheat *TaELF-3* homoeoalleles conferred early heading and the Δ*TaELF-B3* allele showed the greatest effect on the early heading phenotype (Mizuno et al., 2023). In barley (*Hordeum vulgare*), one single nucleotide polymorphism (SNP) causes an amino acid substitution in *ELF3* and leads to plant development acceleration (Zahn et al., 2023). Moreover, an exotic barley *ELF3* allele accelerated inflorescence development at high temperature, which was correlated with early transcriptional induction of MADS-box floral identity genes *BARLEY MADS-box 3* (*BM3*) and *BM8*, and displayed stable total grain number at elevated temperatures (Zhu et al., 2023). In addition, barley core circadian clock genes, *PRR59*, *PRR73*, and *PRR95*, can respond rapidly to temperature in an *ELF3*-dependent manner (Ford et al., 2016). In *Brachypodium distachyon*, FLAVIN-BINDING, KELCH REPEAT, F-BOX 1 (FKF1), ELF3, and Polycomb repressive complex 2 (PRC2) form an FKF1-ELF3-PRC2 regulatory module to control flowering time in response to light, and overexpression of *FKF1* strongly accelerates flowering (Zeng and Qin, 2025).

Common buckwheat is an orphan crop and exhibits heteromorphic self-incompatibility (HSI) resulting from its dimorphic flowers with successful mating only between pin (long pistil and short stamen) and thrum (short pistil and long stamen) (Fawcett et al., 2023; Matsui and Yasui, 2020a; He et al., 2023). It is considered that self-incompatibility, flower morphology, and pollen size are governed by the *S* supergene (Matsui et al., 2003). HSI not only is a major obstacle for establishing pure lines and fixation of agronomically useful genes, but also contributes to low yield in common buckwheat (Matsui and Yasui, 2020b; Jiao et al., 2025). However, buckwheat is more than a gluten-free pseudocereal of high nutritional value and has gained increased attention as an important source of bioactive compounds for function food (Oliveira et al., 2026). *S-ELF3* (*ELF3*-like gene) has been identified as a candidate gene in the S locus and is related to heterostylous self-incompatible (HSI) in common buckwheat (*Fagopyrum esculentum*) (Matsui et al., 2020). Sequence variation of *FhS-ELF3* contributes to the homomorphic self-compatibility of common buckwheat (Zhang et al., 2023). We have developed a self-compatible (SC) common buckwheat line with uniform long homostyle (LH) flowers from a natural mutant individual of buckwheat variety “Bei-zao-sheng”. The mutant individual has flowers with long pistil and long stamen, and legitimate cross-pollinations could occur with pin, thrum, or LH plants. Moreover, an *ELF3* orthologous gene, *FaesELF3*, has been isolated from red flower LH *F. esculentum* and *FaesELF3*-silenced plant produced flower with part of the stamen consisting of short filament and empty anther (male sterile anther) (Ma et al., 2022). In our previous work, downregulation of *FaesMYB15* expression led to lighter tepal color and increased stamen numbers (nine stamens) in red flower LH *F. esculentum* (Liu et al., 2026). *FaesMYB15*-silenced plants and *FaesELF3* overexpression plants in red flower LH common buckwheat show a similar flower phenotype. In this study, combined with yeast one-hybrid (Y1H) assay, dual-luciferase reporter (DLR) assay, and electrophoretic mobility shift assay (EMSA), an R2R3-MYB TF FaesMYB15 was shown to directly bind to the promoter of *FaesELF3* (*pFaseELF3*) and repressed *FaesELF3* expression. In addition, overexpression of *FaesELF3* in red flower LH *F. esculentum* leads to obvious early flowering and rapid growth of plant, and results in flowers with increased tepal and stamen numbers (six tepals and nine stamens) and lighter tepal color. Our study introduces a new regulator module of FaesMYB15-FaesELF3 involved in regulating flowering time, floral color, and floral organ number in plants, providing theoretical support for genetic breeding of flowering time and flowering color in common buckwheat and other crops.

## 2 Materials and methods

### 2.1 Plant material

Red flower LH *F. esculentum* plants were grown under natural conditions in Jingzhou, Hubei Province, China. Inflorescence and flowers were collected from red flower LH common buckwheat at the anthesis stage, and immediately frozen in liquid nitrogen, and stored at −80°C until further analysis.

### 2.2 Yeast one-hybrid assay

Y1H assays were performed using the Yeast One-Hybrid Media Kit (Coolaber, Shanghai, China) according to the manufacturer’s instructions. A 578-bp region (−24/+554) of the *FaesELF3* promoter (*pFaesELF3*) (Ma et al., 2022), containing the R2R3-MYB TF binding sites, was cloned into the pAbAi vector to generate the bait construct (pAbAi-pFaesELF3) with the primers Y1HpFaesELF3F and Y1HpFaesELF3R (**Supplementary Table S1**). The full-length open reading frame (ORF) of *FaesMYB15* (PV476880.1) was cloned into the pGADT7 vector to create the prey construct with primers Y1HFaesMYB15F and Y1HFaesMYB15R (**Supplementary Table S1**). The pAbAi-pFaesELF3 construct was transformed into the Y1HGold competent yeast cells to create bait strains. The prey plasmid pGADT7-FaesMYB15 was introduced into the bait strain for the Y1H assay according to the manufacturer’s instructions. The minimal inhibitory concentration of Aureobasidin A (AbA) for the bait-reporter strains was screened, and the colonies were incubated and selected using methods according to Jiao et al. (2025).

### 2.3 Dual-luciferase reporter assay

DLR assay was performed to investigate whether FaesMYB15 activates or represses *FaesELF3* expression. The 578-bp region (−24/+554) of *pFaesELF3* was cloned into the pGreen0800-LUC vector to create the reporter construct with the primer pairs DualpFaesELF3F and DualpFaesELF3R (**Supplementary Table S1**). In addition, the full-length ORF of *FaesMYB15* was cloned into the pGreenII 62-SK vector to generate the effector construct using the primer pairs DualFaesMYB15F and DualFaesMYB15R (**Supplementary Table S1**). The effector and the reporter *Agrobacterium* cultures were prepared and infiltrated into *N. benthamiana* leaves described obviously (Liu et al., 2026). As a negative control, empty pGreenII 62-SK was transformed with the pGreen0800-pFaesELF3 reporter. The treated tobacco plants were cultured, and the ratio of firefly luciferase (LUC) to renilla luciferase (REN) was assayed according to our previous work (Liu et al., 2026).

### 2.4 Electrophoretic mobility shift assay

The full-length CDS of FaesMYB15 was cloned into the pGEX-6P-1 vector, sequenced, and transformed into *E. coli* strain Rosetta (DE3) for protein expression. The recombinant protein purification and the wild-type probe were marked according to Liu et al. (2026). A 291-bp DNA fragment containing the putative transcription factor binding site (TFBS) was used as the labeled probe, and unlabeled fragments were used as cold competitors. The probes were incubated, competition assays (10-, 50-, and 100-fold unlabeled competitors) were performed, the entire reaction mixture was run, and signals were visualized according to Liu et al. (2026).

### 2.5 Phenotype analysis of 35S::FaesELF3 transgenic red flower LH common buckwheat

The full-length ORF of *FaesELF3* was amplified and cloned into the pHZM27 vector using *XhoI* and *AvrII* restriction sites with the primer pairs TFaesELF3F and TFaesELF3R (**Supplementary Table S1**). The 35S::FaesELF3 construct was transformed into red flower LH common buckwheat using the floral-dip method via *Agrobacterium* cultures. All transgenic seedlings were confirmed by PCR with GFP-specific primer pairs GFP-F and GFP-R and GFP protein observation according to Liu et al. (2026). The 35S::FaesELF3 transgenic red flower LH common buckwheat plants were cultivated under an LEDR-1000 plant growth chamber (Yanghui, Ningbo, China) at 25/20°C day/night temperatures and 70% relative humidity. After flowering, floral phenotypes of the 35S::FaesELF3 transgenic plants were recorded and statistically analyzed. Total RNA was extracted from inflorescences of 35S::FaesELF3 transgenic red flower LH plants and normal red flower LH common buckwheat (CK). First-strand cDNA was then synthesized using the HiScript^®^ II Q RT SuperMix for the qPCR kit (Vazyme, Nanjing, China). The expression of *FaesELF3* in the transgenic flowers and CK flowers was determined by qRT-PCR according to Liu et al. (2026), but with the primer pairs qFaesELF3F and qFaesELF3R (**Supplementary Table S1**).

### 2.6 Comparative transcriptome between inflorescences from 35S::FaesELF3 transgenic red flower LH lines and normal red flower LH common

Inflorescences from 35S::FaesELF3 transgenic red flower LH lines and red flower LH common buckwheat plants (CK) were harvested. Samples were frozen in liquid nitrogen and stored as described above. Each treatment consisted of three independent biological replicates, with three plants randomly selected from each experimental group to represent a single biological replicate. Total RNA was extracted using the RNAprep Pure Plant Plus Kit (Tiangen, Beijing, China). RNA quality and integrity were assessed using a NanoDrop 2000 spectrophotometer (Thermo Fisher Scientific, USA) and an Agilent 2100 Bioanalyzer (Agilent Technologies, USA). High-quality RNA was used for cDNA library construction, and RNA sequencing (RNA-seq) was performed on the DNBSEQ-T7 platform (MGI, Shenzhen, China). Clean reads were then mapped to the reference genome using HISAT2 and transcript assembly was performed using StringTie. Gene expression levels were quantified as fragments per kilobase of transcript per million mapped reads (FPKM). Differentially expressed genes (DEGs) were identified using DESeq2 with a false discovery rate (FDR) < 0.05 and a threshold of |log2FC| ≥ 1. Principal component analysis (PCA) was performed to assess the overall transcriptomic variation among samples. Gene Ontology (GO) and Kyoto Encyclopedia of Genes and Genomes (KEGG) enrichment analyses were subsequently performed to evaluate transcriptomic variation and the biological functions of DEGs. Transcriptome sequencing, bioinformatic analyses, and DEG identification were performed by Smartgenomics Technology Co., Ltd. (Tianjin, China). The raw sequencing data have been deposited in the China National Center for Bioinformation (CNCB) Genome Sequence Archive (GSA) under BioProject accession number PRJCA065629. Moreover, expressions of *FaesELF3* and its target genes were separately detected in the flowers of 35S::FaesELF3 transgenic plants by using qRT-PCR method described above.

### 2.7 Measurement of total anthocyanin content in inflorescence of 35S::FaesELF3 transgenic plants and normal red flower LH common buckwheat

Total anthocyanin content was determined using a pH differential spectrophotometric assay with minor modifications (Niu et al., 2010). Inflorescences of 35S::FaesELF3 transgenic lines and normal red-flowered LH common buckwheat were collected, immediately frozen in liquid nitrogen, and powdered. Approximately 1 g of tissue was extracted with 5 mL of 0.05% HCl in methanol at 4°C for 12 h. After centrifugation at 10,000×*g* for 20 min, the residue was re-extracted with 5 mL of extraction solution for 6 h. The combined supernatants were used for anthocyanin determination. Subsequently, 1 mL of the combined supernatant was mixed with 4 mL of either buffer A (0.4 M KCl, pH adjusted to 1.0 using 2 N HCl) or buffer B (1.2 N citric acid, pH adjusted to 4.5 with 0.2 M NaH_2_PO_4_), and absorbance values were recorded at 510 and 700 nm. The anthocyanin content was calculated according to Romero et al. (2008) using the following formula: TA = *A* × MW × 5 × 100 × *V*/ϵ. In this equation, TA denotes the total anthocyanin content (mg/100 g), *A* is calculated as (A510–A700) pH 1.0 – (A510–A700) pH 4.5, *V* is the final extract volume (mL), MW is the molecular weight of cyanidin-3-O-glucoside (449.2 g/mol), and ϵ is the molar absorptivity (26,900).

## 3 Results

### 3.1 FaesMYB15 represses *FaesELF3* transcription via directly binding to *pFaesELF3*

Previous studies suggested that the MYBCORE (TTAGG-motif), TAGGT-motif, and TTGTT-motif were key cis-acting elements for R2R3-MYB TF recognition and action (Franco-Zorrilla et al., 2014). In order to assay whether our previously identified R2R3-MYB TF FaesMYB15 directly binds the *FaesELF3* promoter regions containing TTAGG-motif, TAGGT-motif, or TTGTT-motif or not, Y1H assay, DLR assay, and EMSA were introduced. Y1H assays demonstrated that FaesMYB15 directly binds to the *pFaesELF3* region (−24/+554) (**Figure 1A**). DLR further confirmed that FaesMYB15 downregulates *FaesELF3* expression in plants (**Figure 1B**). Furthermore, the presence of FaesMYB15 resulted in a significant decrease in the LUC/REN fluorescence ratio compared to the control (*p <* 0.05) (**Figure 1C**). Moreover, EMSA confirmed that FaesMYB15 could bind to the *FaesELF3* promoter region. A shift band was observed with labeled probe incubated with FaesMYB15 recombinant protein, whereas this shift band disappeared when a 100-fold competitor was added (**Figures 1D, E**). In addition, the *FaesELF3* expression level decreased significantly in the flowers of pFaesMYB15::FaesMYB15 transgenic LH *F. esculentum* (**Supplementary Figure S1**). These observations suggested that FaesMYB15 directly represses *FaesELF3* to regulate floral development in *LH F. esculentum*.

**Figure 1:**
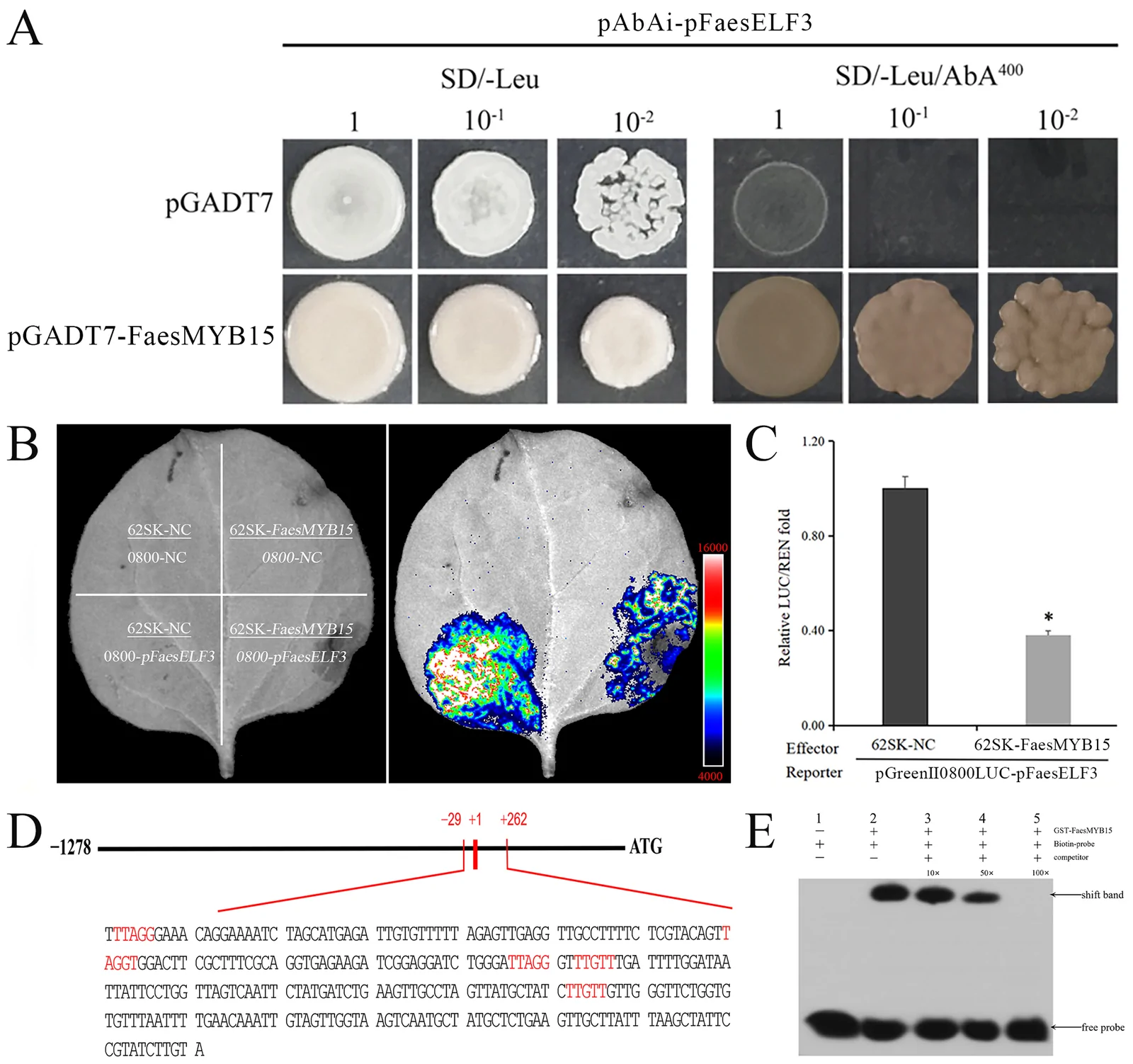
FaesMYB15 directly binds to the *FaesELF3* promoter and represses expression of *FaesELF3*. **(A)** Y1H assay suggests that FaesMYB15 directly binds to the *FaesELF3* promoter. pGADT7-FaesMYB15 as the prey construct, pAbAi-pFaesELF3 as the bait construct. The bait construct was co-transformed with either the prey construct or the empty pGADT7-AD vector into Y1HGold yeast cells. Transformants were grown on SD/-Leu medium in the absence (left) or presence (right) of 400 ng mL^−1^ AbA. **(B)** Representative images of the DLR assay in *N. benthamiana* leaves. **(C)** DLR assay indicates that FaesMYB15 represses *FaesELF3* expression determined by the LUC/REN ratio. Statistical significance was determined using ANOVA with LSD test. *p <* 0.05. **(D)** Distribution of key cis-acting elements for R2R3-MYB TFs binding in the *FaesELF3* promoter. **(E)** EMSA suggests that FaesMYB15 directly bind to *pFaesELF3*. “+” and “−” indicate the presence and absence of the corresponding components, respectively. The 10-fold, 50-fold, and 100-fold concentrations of cold probes were used for competition, respectively. Arrows indicate the positions of the shifted and free probes.

### 3.2 Phenotype analysis of 35S::FaesELF3 transgenic red flower LH common buckwheat

To investigate the function of *FaesELF3* involved in floral development of red flower LH common buckwheat, the 35S::FaesELF3 construct was transformed into red flower LH common buckwheat via *Agrobacterium*-mediated transformation. All transgenic seedlings were verified by PCR and GFP protein observation. Four independent transgenic lines of 35S::FaesELF3 LH common buckwheat were obtained. Among four 35S::FaesELF3 transgenic red flower LH common buckwheat lines, one (25%) independent 35S::FaesELF3 transgenic plant (35S::FaesELF3-L1) produced flowers with increased tepal numbers (six tepals) (**Figure 2A**, Type I), one (25%) independent transgenic line (35S::FaesELF3-L2) produced flowers with increased tepal numbers (six tepals) and a lighter tepal color (**Figure 2A**, Type II), and two (50%) independent transgenic lines (35S::FaesELF3-L3 and 35S::FaesELF3-L4) produced flowers with increased tepal numbers (six tepals), increased stamen numbers (nine stamens), and a lighter tepal color (**Figure 2A**, Type III).

**Figure 2:**
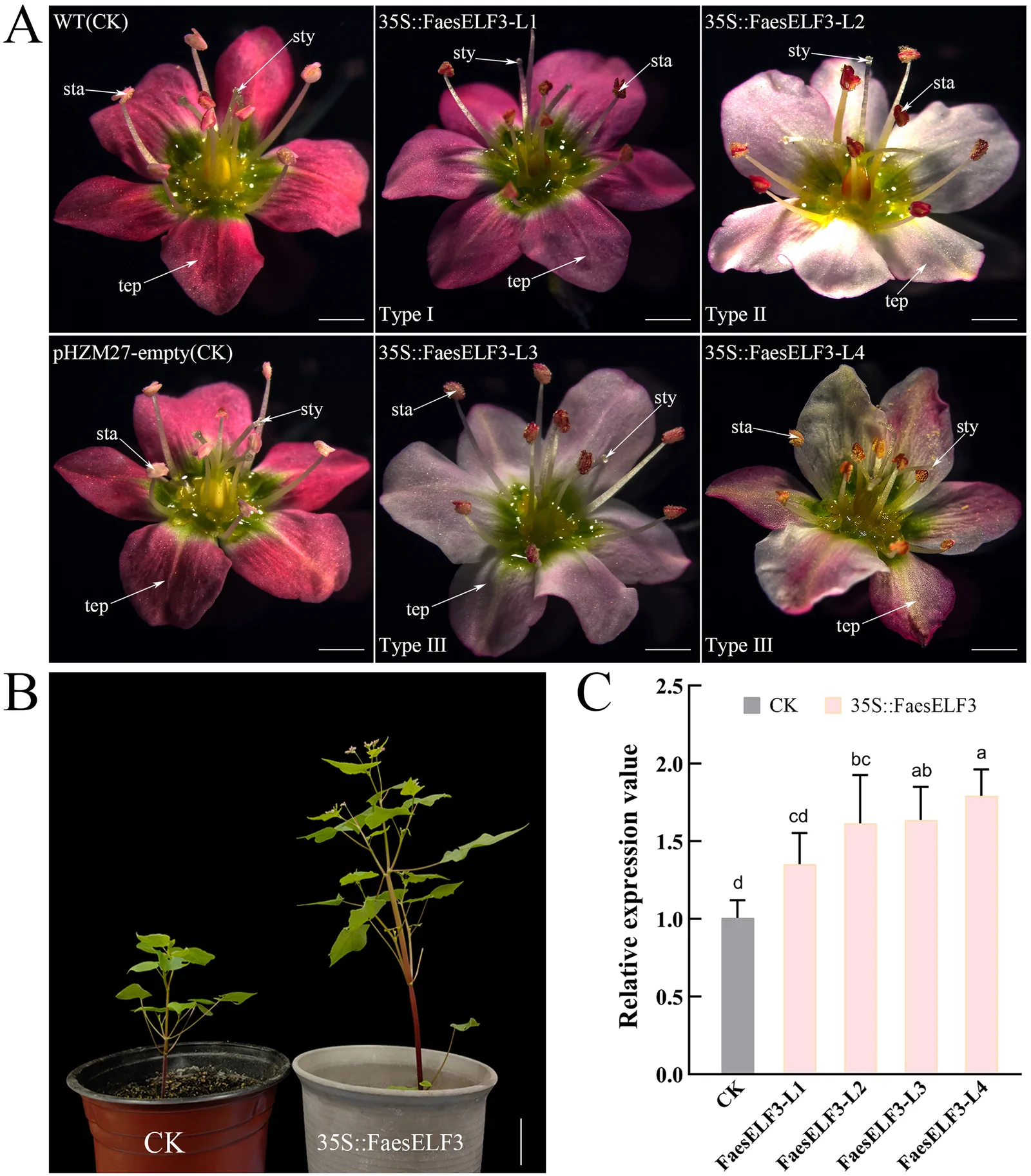
Phenotypic changes of 35S::FaesELF3 transgenic red flower LH common buckwheat. **(A)** Flower phenotype comparison between normal red flower LH plant, pHZM27-empty transgenic red flower LH plant, and 35S::FaesELF3 transgenic red flower LH common buckwheat. WT (CK): normal flower of red flower LH plants with five tepals (tep), eight stamens (sta), and three styles (sty); CK: flower of pHZM27-empty transgenic plant show similar phenotype with WT; Type I: 35S::FaesELF3-L1 flower with tepal number increased (six tepals); Type II: 35S::FaesELF3-L2 flower with tepal number increased (six tepals) and lighter tepal color; Type III: 35S::FaesELF3-L3 and 35S::FaesELF3-L4 flowers with increased tepal number (six tepals), increased stamen number (nine stamens), and lighter tepal color (scale bar: 1 mm). **(B)** 35S::FaesELF3 transgenic plant showing obviously earlier flowering and rapid growth than normal red flower LH plant (scale bar: 5 cm). **(C)** Comparison of expression levels of *FaesELF3* in flowers between CK and four lines of 35S::FaesELF3 transgenic LH *F. esculentum*.

Compared with normal red flower LH common buckwheat, the four 35S::FaesELF3 transgenic lines exhibited an earlier flowering phenotype and rapid growth of plant (**Figure 2B**). The flowering dates of all the transgenic lines show 5–8 days earlier than the normal red flower LH plants (CK). Moreover, the expression level of *FaesELF3* was significantly increased in all independent 35S::FaesELF3 transgenic lines with flower phenotype change (*p <* 0.05, LSD). Among the four transgenic lines, the highest expression level (*p <* 0.05, LSD) of *FaesELF3* was observed in the 35S::FaesELF3-L4, which produced flowers with increased tepal and stamen numbers, but a lighter tepal color. The lowest expression level of *FaesELF3* was detected in the 35S::FaesELF3-L1 that produced flowers with increased tepal numbers and without tepal-color changes (**Figure 2C**).

### 3.3 Comparative transcriptome between inflorescences of 35S::FaesELF3 transgenic red flower LH lines and normal red flower LH common buckwheat

The RNA-seq technique was employed to compare the differences in transcriptome profiles between 35S::FaesELF3 transgenic lines and normal LH common buckwheat to explore the transcriptional changes resulting from *FaesELF3* overexpression. PCA showed that the samples of 35S::FaesELF3 transgenic lines revealed a clear separation with the LH common buckwheat, indicating distinct transcriptomic profiles and high reproducibility among biological replicates (**Figure 3A**). The DEGs included 2,157 upregulated and 2,417 downregulated genes (**Figure 3B**). Then, with the trend clustering analysis, the subset of consistently downregulated genes were identified in 35S::FaesELF3 transgenic lines (**Figure 3C**). GO enrichment analysis of the downregulated DEGs revealed significant enrichment of biological processes related to cell wall organization, cell wall modification, and external encapsulating structure organization. Enriched molecular functions included pectinesterase (PE) activity and calcium ion binding, whereas enriched cellular components were associated with the cell periphery and extracellular region (**Figure 3D**). In addition, we further performed KEGG enrichment analysis of the upregulated genes, which revealed enrichment of the plant circadian rhythm pathway (**Supplementary Figure S2A**). This observation is consistent with the role of *ELF3* as a core component of the plant circadian clock. Moreover, the expression levels of upregulated genes involved in flowering regulation (such as *SOC1*-like *FaesSOC1* and *FT*-like *FaesFT*) were increased in the 35S::FaesELF3 transgenic plants (**Supplementary Figure S2B**).

**Figure 3:**
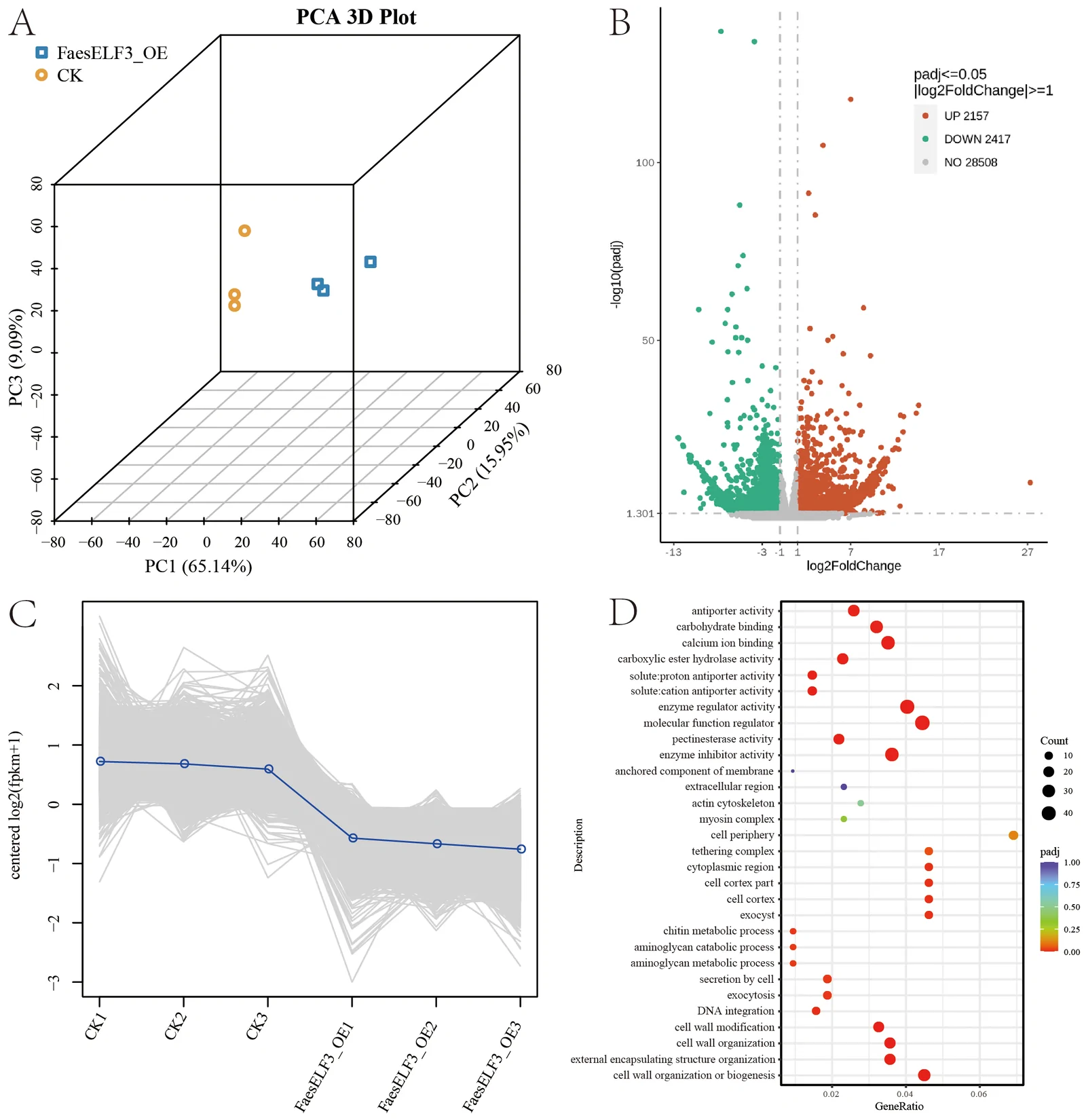
Comparative transcriptome between 35S::FaesELF3 transgenic lines and normal red flower LH common buckwheat. **(A)** PCA of transcriptome data from 35S::FaesELF3 overexpressing lines (FaesELF3_OE) and normal red flower LH plants (CK). **(B)** Volcano plot of DEGs, with red dots representing upregulated genes, green dots representing downregulated genes, and gray dots representing non-significantly expressed genes. **(C)** Trend clustering analysis of DEGs; blue line represents the average expression profile and gray lines represent individual gene expression profiles. **(D)** GO enrichment analysis of DEGs in 35S::FaesELF3 transgenic lines compared with normal red flower LH plants. The top enriched GO terms from the biological process, cellular component, and molecular function categories are shown.

Heatmap analysis showed that *FaesELF3* overexpression markedly downregulated the expression of genes associated with pseudo-response regulator (PRR), pectin metabolism, AGAMOUS (AG)-like MADS-box protein, and calcium signaling (**Figure 4A**). Among these, multiple genes encoding PRRs were consistently downregulated in the 35S::FaesELF3 transgenic lines compared with the normal LH common buckwheat (CK). Similarly, numerous PE genes exhibited reduced transcript abundance. In addition, several *AG*-like MADS-box genes were consistently downregulated. Genes associated with calcium signaling, including members of the *CAM*, *CBP*, *CDPK*, and *CML* gene families, also displayed broadly reduced expression in the transgenic plants. The expression patterns of these genes were highly consistent among biological replicates, demonstrating the reliability and reproducibility of the transcriptome data. To validate the RNA-seq results, eight representative genes, namely, three PRR genes (*FaesPRR5-1*, *FaesPRR5-2*, and *FaesPRR95*), one PE gene (*FaesPE21-4*), two AG-like MADS-box genes (*FaesAGL18* and *FaesAGL19*), a calcium-dependent protein kinase gene (*FaesCDPK28*), and a calcium-binding protein gene (*FaesCML23*), were selected for qRT-PCR. The results indicated that the expression of each gene was downregulated in the 35S::FaesELF3 transgenic lines (**Figure 4B**), which was consistent with the transcriptome data.

**Figure 4:**
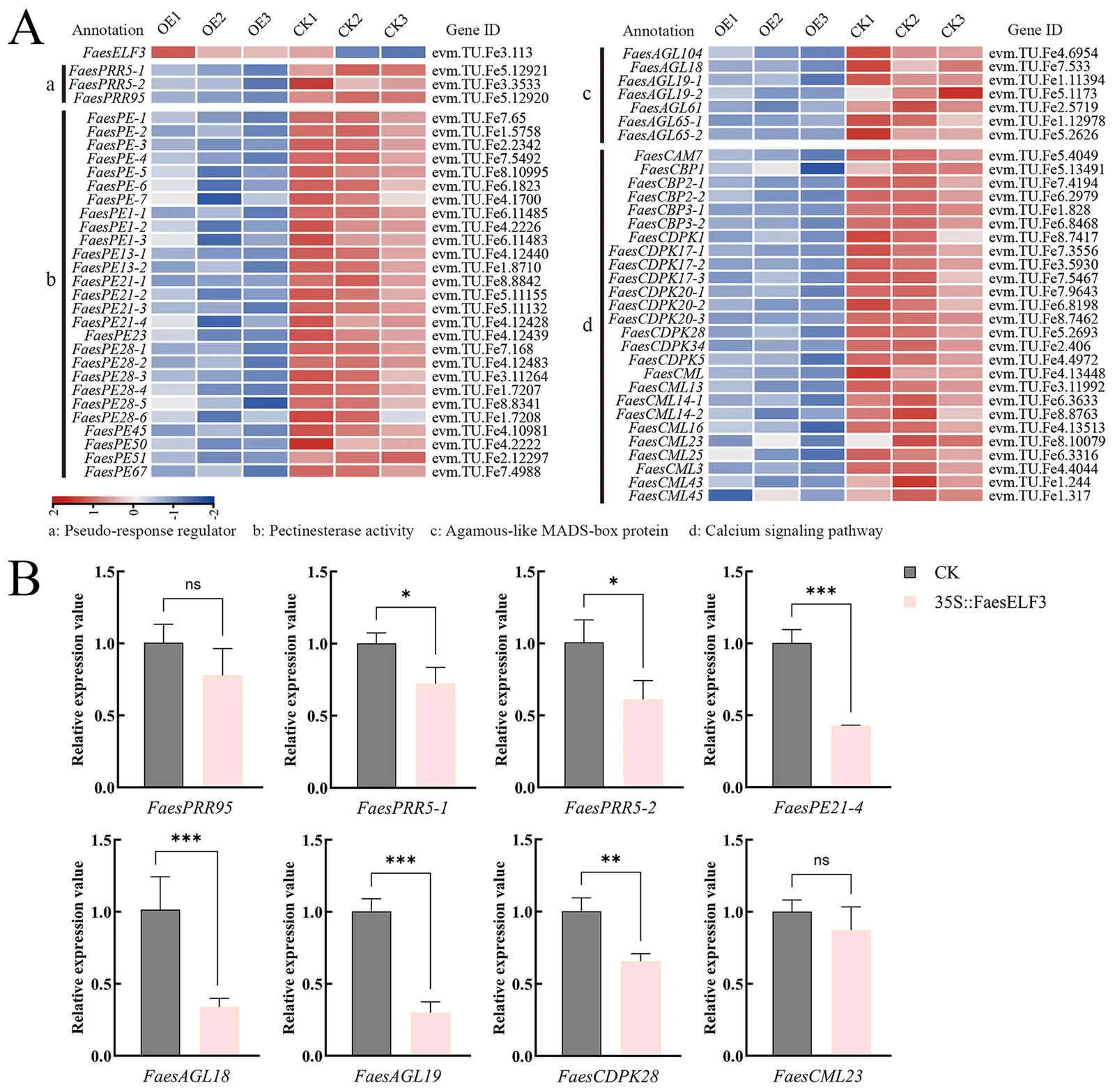
Expression analysis of candidate genes involved in flowering time in 35S::FaesELF3 transgenic red flower LH common buckwheat. **(A)** Heatmap showing the expressions of candidate genes involved in flowering time in flowers of 35S::FaesELF3 transgenic plants (OE) and normal red flower LH common buckwheat (CK). **(B)** Comparison of relative levels of some candidate genes between 35S::FaesELF3 transgenic lines and red flower LH common buckwheat (CK) determined by qRT-PCR. Asterisks (*), double asterisks (**), and triple asterisks (***) indicate a statistical significance at *p <* 0.05, *p <* 0.01, and *p <* 0.001, respectively.

### 3.4 Overexpression of *FaesELF3* inhibits anthocyanin accumulation in tepal of red flower LH common buckwheat

35S::FaesELF3 transgenic plants produced flowers with an obviously lighter tepal color. KEGG enrichment analysis of the downregulated DEGs further revealed significant enrichment of two pathways associated with anthocyanin biosynthesis, namely, the phenylpropanoid biosynthesis and flavonoid biosynthesis pathways (**Figure 5A**). Previous reports have shown that *CHS* and *F3H* are key enzymes in the flavonoid biosynthetic pathway, whereas *LDOX/ANS* catalyzes a late step leading to anthocyanidin formation (Li et al., 2010; Wang et al., 2021). GST proteins are involved in the intracellular transport of anthocyanins, and *FeGST1* has previously been associated with anthocyanin accumulation in common buckwheat (Matsui et al., 2018). In this study, heatmap analysis revealed that genes involved in anthocyanin biosynthesis and transport were markedly downregulated in the 35S::FaesELF3 transgenic lines compared with CK (**Figure 5B**). These genes included the anthocyanin biosynthetic structural genes *FeCHS2*, *FeF3H*, *FeLDOX*, and multiple *FeGST* family genes related to anthocyanin transport. Moreover, total anthocyanin content in inflorescence of each 35S::FaesELF3 line was significantly lower than that in normal red flower LH common buckwheat (**Figure 5C**). These results suggest that the lighter tepal color phenotype observed in 35S::FaesELF3 red flower LH common buckwheat is associated with the transcriptional changes of anthocyanin biosynthesis-related genes and the decreased anthocyanin accumulation.

**Figure 5:**
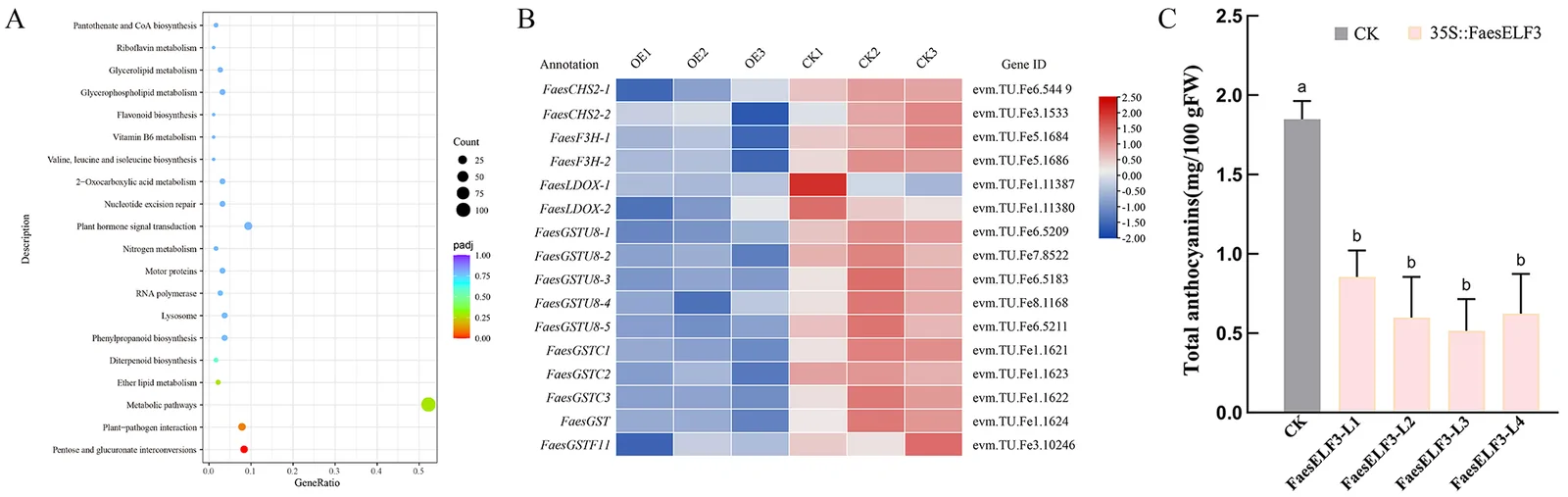
Anthocyanin biosynthesis analysis in 35S::FaesELF3 transgenic red-flowered LH common buckwheat. **(A)** KEGG enrichment analysis of downregulated DEGs in inflorescences of 35S::FaesELF3 transgenic plants compared with normal red flower LH plants. Dot size indicates gene count, and dot color represents the adjusted *p*-value. **(B)** Heatmap showing the expressions of anthocyanin biosynthesis pathway genes involved in flowering time in flowers of 35S::FaesELF3 transgenic lines (OE) and normal red flower LH common buckwheat (CK). **(C)** Total anthocyanin content in inflorescences of CK and four independent 35S::FaesELF3 transgenic lines. Data are shown as mean ± SD. Different lowercase letters indicate significant differences (*p <* 0.05).

## 4 Discussion

### 4.1 *FaesELF3* promotes flowering and rapid growth of plant in LH common buckwheat

The EC, consisting of the ELF3, ELF4, and LUX proteins, is an essential component of the circadian clock, and binds to the *PIF4* and *PIF5* gene promoters and suppresses expression of these genes during early night (Legris et al., 2017). *Arabidopsis ELF3* represses expression of *PRR9* and *PRR7* to regulate circadian clock dynamics and different aspects of plant development, and was essential for hypocotyl development (Yuan et al., 2024). In *Arabidopsis*, AGL6 directly repressed ELF3, which, in turn, directly suppressed FT, and the three TFs formed AGL6-ELF3-FT cascade regulatory module to control flowering time (Lee et al., 2024). *FT*, *SUPPRESSOR OF OVEREXPRESSION OF CONSTANS1* (*SOC1*), and *LEAFY* (*LFY*) are floral integrators, which were repressed by *ELF3*, and *GAI ASSOCIATED FACTOR 1* (*GAF1*) forms a transcriptional repressor complex and promotes the expression of *FT* and *SOC1* through the repression of *ELF3* in the GA-dependent flowering pathway (Fukazawa et al., 2021). However, rice *OsELF3–1* affects the circadian system and acts as a floral promoter under both short-day and long-day conditions (Sun et al., 2024). Moreover, *Arabidopsis* SOC1 and FT function as crucial integrators that promote flowering (Huang et al., 2025; Gao et al., 2026). In this study, *FaesSOC1* and *FaesFT* expression increased obviously in 35S::FaesELF3 transgenic plants. In *B. distachyon*, the FKF1-ELF3-PRC2 regulatory module controls flowering time in response to light via regulating the flowering activator *PPD1* and is likely conserved across temperate grasses (Zeng and Qin, 2025). Wheat *ELF3* operates downstream of *PHYB* as a direct transcriptional repressor of *PPD1*, which further regulates *FT1* expression, and regulates wheat heading time in response to photoperiod (Alvarez et al., 2023). In addition, plant-specific gene *GIGANTEA* (*GI*) accelerates wheat heading time by promoting *FT1* expression via interactions with *ELF3*, *CONSTANS* (*CO*), and the age-dependent microRNA172-*APETALA2* (*AP2*) pathway (Li et al., 2024). Moreover, wheat *ELF3* homolog can affect heading date separately from effects on the circadian oscillator, suggesting that the structure of the wheat circadian oscillator might differ from that of *Arabidopsis* (Wittern et al., 2023). EC directly binds and suppresses the expression of flowering repressors *PRR37* and *Ghd7*, and integrates photoperiod signals to control flowering in rice (Andrade et al., 2022). A similar result was also observed in our study: the *FaesPRR5–1* and *FaesPRR5–2* expression was significantly downregulated in 35S::FaesELF3 transgenic LH common buckwheat (**Figure 4B**). Moreover, overexpression of *FaesELF3* leads to obvious early flowering and rapid growth in transgenic lines. These suggest that the ELF3-PRR pathway involved in regulating flowering is evolutionarily conserved in common buckwheat.

In rice, E3 ubiquitin ligase IPA1-interaction protein 1 (IPI1) controls flowering by targeting *OsELF3-1* (Xu et al., 2024). In chickpea (*Cicer arietinum*), the 11-bp deletion in the first exon of *ELF3* homolog was associated with early flowering (Ridge et al., 2017). A novel exotic *ELF3* allele resulted from one SNP causing an amino acid substitution (W669G) and accelerated plant development compared with the cultivated *ELF3* allele in barley (Zahn et al., 2023). In hybrid aspen (*Populus tremula* × *tremuloides*), *ELF3* ortholog acts as a key mediator of low temperature (LT)-induced growth cessation, and also functions in short photoperiod (SP)-mediated growth cessation by downregulating *FT2* in leaves (Nair et al., 2025). In 35S::FaesELF3 transgenic LH common buckwheat, *FaesPE21-4*, *FaesAGL18*, *FaesAGL19*, and *FaesCDPK28* involved in regulating flowering are also significantly downregulated.

### 4.2 A novel transcriptional regulatory module of FaesMYB15-FaesELF3 involved in floral organ number, flower color, and flowering in plant

In maize (*Zea mays*), ZmELF3.1 forms the EC through interacting with ZmELF4 and ZmLUX to regulate flowering, and ZmELF3.1 directly interacts with both RA2 and ZmELF4.2 to form the ELF3–ELF4–RA2 complex that further enhances the binding of RA2 to the promoter of *TSH4*, leading to suppressed *TSH4* expression and consequently decreased Tassel branch number (TBN) (Xie et al., 2024). Barley plants carrying an exotic *ELF3* allele displayed stable total grain number at elevated temperatures (Zhu et al., 2023). Truncation mutations in the A- and B-genome copies of *ELF3* flowered earlier and had less spikelets per spike than the wild-type control under short- and long-day conditions in wheat (Alvarez et al., 2016). All these results suggested that *ELF3* homologs have evolved to regulate floral organ number in different plants. In this study, overexpression of *FaesELF3* in red flower LH *F. esculentum* led to an increase in tepal number (six tepals), an increase in stamen number (nine stamens), and a lighter tepal color. In our previous work, an R2R3-MYB FaesMYB15-silenced results in increased stamen numbers (nine stamens) and lighter tepal color of red color flower (Liu et al., 2026), which shows a similar phenotype to the 35S::FaesELF3 transgenic red flower LH common buckwheat. Moreover, recent studies suggested that R2R3-MYB TFs were involved in regulating flower color in many plants via activating anthocyanin biosynthesis (Wang et al., 2025; Yu et al., 2026; Ye et al., 2026). In the present study, the expression of genes related to anthocyanin biosynthesis and transport in 35S::FaesELF3 transgenic plants was downregulated, and total anthocyanin content in inflorescences of 35S::FaesELF3 lines was significantly lower than that of the normal red flower LH common buckwheat (CK). In addition, Y1H assay, DLR assay, and EMSA further suggested that FaesMYB15 directly binds to the *FaesELF3* promoter and suppresses *FaesELF3* expression. Our findings propose that a novel regulatory module of FaesMYB15-FaesELF3 mediates floral organ number, flower color, and flowering in plant (**Figure 6**).

**Figure 6:**
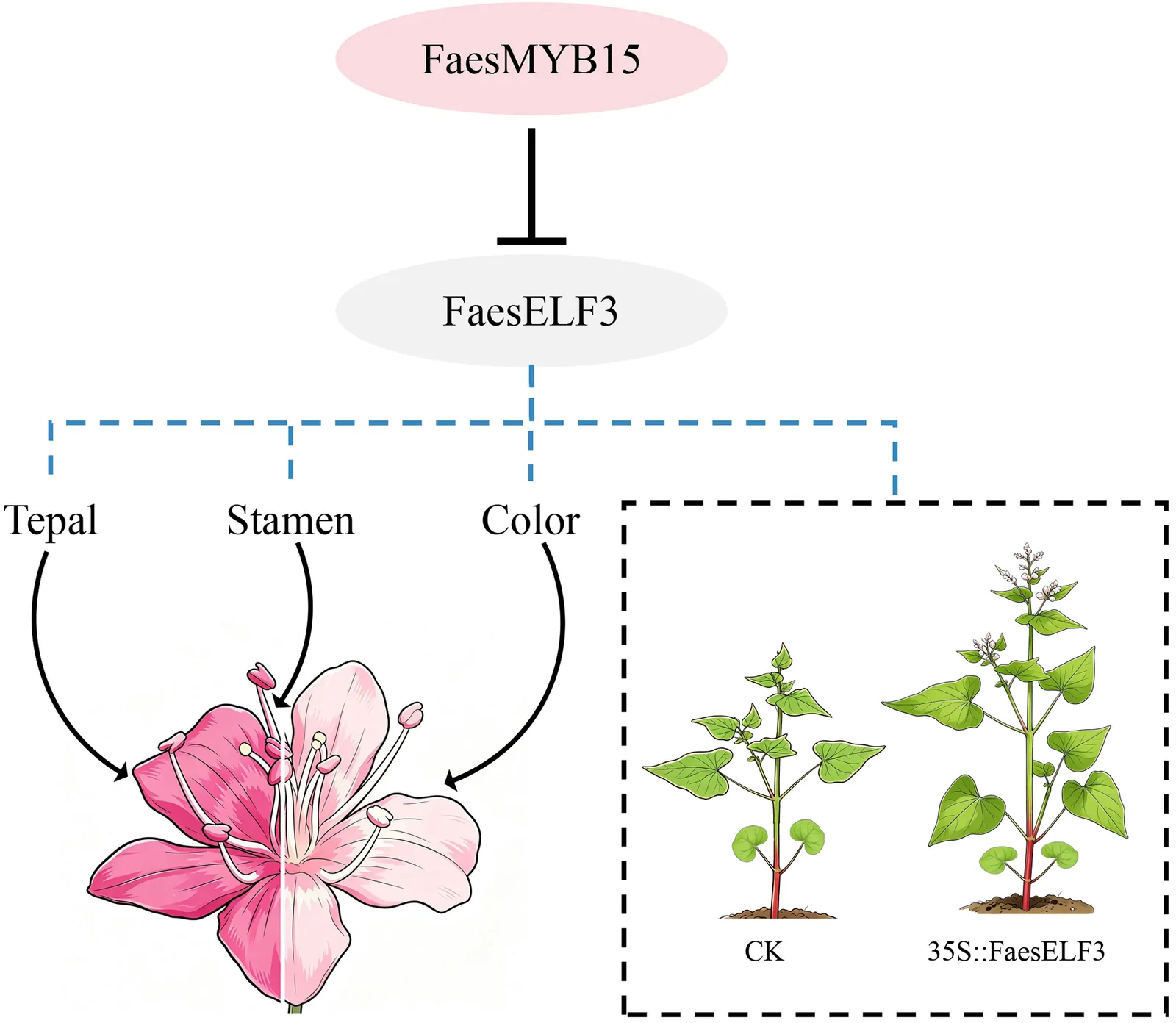
The FaesMYB15-FaesELF3 module involved in regulating flowering time, floral organ number, and floral color in red flower Long-homostyle common buckwheat.

## 5 Conclusion

This study aimed to elucidate how the circadian clock component *FaesELF3* regulates flowering time and flower development in red flower LH common buckwheat. Y1H assay, DLR assay, and EMSA suggested that R2R3-MYB TF FaesMYB15 directly binds to the *FaesELF3* promoter and suppresses expression of *FaesELF3*. In addition, overexpression of *FaesELF3* in 35S::FaesELF3 transgenic red flower LH *F. esculentum* leads to obvious early flowering and rapid growth of plant, and results in flowers with increased tepal numbers and stamen numbers and a lighter tepal color. Moreover, total anthocyanin content in inflorescence of the 35S::FaesELF3 line was significantly lower than that of the normal red flower LH common buckwheat (CK). *FaesELF3* overexpression markedly downregulates the expression of two common buckwheat core circadian clock genes, *FaesPRR5–1* and *FaesPRR5-2*, and two AG-like MADS-box genes, *FaesAGL18* and *FaesAGL19*, involved in flowering time. Our study introduces a new regulator module of FaesMYB15-FaesELF3 involved in flowering time, floral color, and floral organ number in plants, and also provides new insights for genetic breeding of flowering time and flower color in common buckwheat.

## Data Availability

The raw sequencing data have been deposited in the China National Center for Bioinformation (CNCB) Genome Sequence Archive (GSA) under BioProject accession number PRJCA065629.
