# Supplementary material for: The FaesMYB15-FaesELF3 module regulates flowering time, floral organ number, and floral color in red flower long-homostyle common buckwheat
Source: Front Plant Sci. 2026 Oct 28;17:1944721. doi: 10.3389/fpls.2026.1944721 (PMC13752136; doi:10.3389/fpls.2026.1944721)
Supplement: Supplementary file 1 [file DataSheet1.doc]

# **Supplementary material**

**Supplementary Table S1:** List of primer sequences used in this study

| **Primers for Yeast One-Hybrid Assay** | | |
| --- | --- | --- |
| **Primer name** | **Primers sequences (5'-3')** | |
| Y1HpFaesELF3F | AAGCTTGAATTCGAGCTCGGAAACAGGAAAATCTAGCAT | |
| Y1HpFaesELF3R | ACATGCCTCGAGGTCGACTCAACCTCTCTTAACAATCCA | |
| Y1HMYB15F | ATGGAGGCCAGTTCTAGAAGAAATGGGTCGATCTCCA | |
| Y1HMYB15R | ATTCATCTGCAGCTCGAGCTCAACCACAACTCTCCAAGAT | |
| **Primers for Dual-Luciferase Reporter Assay** | | |
| **Primer name** | **Primers sequences (5'-3')** | |
| Dual-pFaesELF3F | GGGCCCCCCCTCGAGGTCGACGGAAACAGGAAAATCTAGCAT | |
| Dual-pFaesELF3R | CAGGAATTCGATATCAAGCTTTCAACCTCTCTTAACAATCCA | |
| Dual-FaesMYB15F | CGCGGTGGCGGCCGCTCTAGATTGAGAGGTTTTGAGAAATGGG | |
| Dual-FaesMYB15R | AAGCTTGATATCGAATTCTAAACCACAACTCTCCAAGATC | |
| **Overexpressed construction Primer** | | |
| Primer name | | Primers sequences (5'-3') |
| TFaesELF3-F | | GAGAGGACACGCTCGAGCCTGTGCCTTTGTTGGATTGTTA |
| TFaesELF3-R | | CCATGAGCTCCCTAGGCCACGGATGATTTAACTCGCTAG |
| **GFP validate Primer** | | |
| Primer name | | Primers sequences (5'-3') |
| GFP-F | | GACGTAAACGGCCACAAGTT |
| GFP-R | | GAACTCCAGCAGGACCATGT |
| **Primers for qRT-PCR** | | |
| Primer name | Primers sequences (5'-3') | |
| qFaesactinF | ACCTTGCTGGACGTGACCTTAC | |
| qFaesactinR | CCATCAGGAAGCTCATAGTTC | |
| qFaesELF3F | CGGAAAGAGGAGGGCCAAGAG | |
| qFaesELF3R | GACGCGTATTTCCTGGGTTGT | |
| qFaesAGL18F | TTGAAGAAGGCTAAAGAACT | |
| qFaesAGL18R | TTCGATTTCTCTACTAGGGG | |
| qFaesAGL19F | GGAGGGAAACCAAGTGATGA | |
| qFaesAGL19R | TCTTCCATTGAGCATGAATC | |
| qFaesPE21-4F | TGCCAGTACAGCCACCTTGA | |
| qFaesPE21-4R | GACAGATCTCGAGGGCCTGG | |
| qFaesPRR5-1F | GGGCGATTTGATGGTGCTTG | |
| qFaesPRR5-1R | GAATCATCAGCTTCCACCAA | |
| qFaesPRR5-2F | CCAAGAGAAGAGGAGTGCTT | |
| qFaesPRR5-2R | TCCAGAGAGACCTTGAGACT | |
| qFaesPRR95-F | GCCTCTGCCTTTTCACGTT | |
| qFaesPRR95-R | CCTCATCTATATCGCAAGCAC | |
| qFaesCDPK28F | ATGGGAATCTGTACGTCCAC | |
| qFaesCDPK28R | AAGTCGTCGCATTCATGATC | |
| qFaesCML23F | CTCCAACGGTGACGGACTAG | |
| qFaesCML23R | CATAAGCCTAAACGCCTCCT | |

**Supplementary Figure.S1:** Expression analysis of *FaesELF3* in pFaesMYB15::FaesMYB15 transgenic LH *F. esculentum*.

**Supplementary Figure.S2:** Heatmap showing the expressions of anthocyanin biosynthesis pathway genes involved in flowering time in flowers of 35S::FaesELF3 transgenic lines (OE) and normal red flower LH common buckwheat (CK) respectively.
